# Empowered by Intertwined Theory and Practice – Experiences From a Diabetes Sports Camp for Physically Active Adults With Type 1 Diabetes

**DOI:** 10.3389/fcdhc.2021.655238

**Published:** 2021-07-15

**Authors:** Stig Mattsson, Peter Adolfsson, Johan Jendle, Viktor Bengtsson, Carina Sparud-Lundin

**Affiliations:** ^1^ Institute of Medical Sciences, Örebro University, Örebro, Sweden; ^2^ Diabetes Endocrinology and Metabolism Research Center, Örebro University, Örebro, Sweden; ^3^ Institute of Clinical Sciences, Sahlgrenska Academy at University of Gothenburg, Gothenburg, Sweden; ^4^ Department of Pediatrics, The Hospital of Halland, Kungsbacka, Sweden; ^5^ Department of Pediatric Medicine, The Queen Silvia Children’s Hospital, Gothenburg, Sweden; ^6^ Institute of Health and Care Sciences, Sahlgrenska Academy at University of Gothenburg, Gothenburg, Sweden

**Keywords:** diabetes type 1, diabetes self-management, diabetes sports camps, physical exercise, experiences, interviews

## Abstract

**Aims:**

To describe the experiences of individuals with diabetes type 1 (T1D) participating in diabetes sports camps and how acquired knowledge could be used in daily self-management.

**Methods:**

Semi-structured telephone interviews were conducted with 15 adults with T1D. A strategic sample procedure was chosen. The interviews were analyzed using qualitative content analysis.

**Results:**

The overarching theme ”Empowered by intertwined theory and practice”, included three main categories: Learning in a motivation-enhancing environment, incorporation of new habits and perceptions of glycemic control and health-related outcomes. The participants considered the camp to be an excellent opportunity to share feelings, ideas, and knowledge. They felt empowered by the camp atmosphere as well as supportive environment. After the camp, the general well-being was improved by incorporating new habits and improvements in glucose control.

**Conclusions:**

A diabetes sports camp constitutes an excellent, but resource-intensive, complimentary support in diabetes care and provides opportunities for T1D individuals to become more independent and autonomous. The findings indicate the need for more directed learning activities for individuals with type 1 diabetes and health care providers to increase their competence in the area of T1D and exercise in order to adequately manage counseling in various types of sports.

## Introduction

Well-controlled diabetes can delay or prevent the development of diabetes complications (1). The effort involved in achieving good glycemic control places high demands on most people with type 1 diabetes (T1D). Physical exercise (PE) is of fundamental importance for both health and well-being (2) and is recommended (along with diet and insulin) as one of the cornerstones in the treatment of T1D (3). However, PE is associated with an increased incidence of both hypo- and hyperglycemia (4). In T1D, hypoglycemia is considered to be a major barrier to PE and the main reason for deteriorated glucose control (5). In daily practice for people with diabetes severe fluctuations in glucose on days with PE is a common phenomenon. The increased glucose variability is often associated with frustration, as satisfactory glycemic control becomes difficult to achieve.

Achieving stable glucose regulation in relation to PE of different duration and intensity puts high demands on appropriate insulin adjustments and adapted carbohydrate intake (6). For physically active T1D individuals, a high degree of educational support is therefore required (7). Education and increased self-perceived knowledge in the area of T1D have been shown to have a major impact on an individual’s ability to manage their diabetes self-care (7). Adolfsson et al. showed that education and feedback related to PE resulted in improved glucose control and increased levels of physical activity (8). It has been observed that individuals with T1D who increase their knowledge of nutrition and insulin adjustments to minimize PE-induced hypoglycemia also reduce their barrier to physical activity (5). However, most patients with poor glycemic control have already received diabetes self-management education (glucose testing, bolus calculation, insulin adjustments in relation to PE etc.) but failed to implement this into their daily regimen (7). There can be many different reasons for not implementing learned self-management routines: difficulties in achieving glycemic goals, fear of hypoglycemia, feelings of failure or lack of support (9). A fundamental problem regarding adequate counseling on sport and T1D is that health care professionals (HCPs) often lack sufficient knowledge (10). The American Diabetes Association (ADA) suggests that patients with T1D may benefit from working with a physiologist experienced in diabetes and exercise to adequately assist them in formulating a safe and effective exercise prescription (3).

Diabetes camps with multidisciplinary participation, including physicians, nurses and dietitians, have been proven to contribute to learning principles of self-management positively (11). Diabetes sports camps can thus enable theoretical and practical learning of principles related to the specifics of PE. Moreover, such camps offer an excellent opportunity to share and discuss experiences. Increased understanding of the participants’ experiences of structured education and support in T1D and PE can provide enhanced knowledge, which may lead to more targeted and tailored healthcare efforts for physically active patients with T1D who regularly perform PE. Our research group arranged eight sports camps for individuals with T1D, resulting in significant improvements of self-estimated knowledge in the area of insulin adjustments and carbohydrate intake, measured directly after the camp. Furthermore, improved glucose control was confirmed, measured as glycated hemoglobin (HbA1c), 3 and 12 months after the sports camp (12). This study aimed to further explore the mechanisms behind these results by describing the individual experiences gained during a sports camp, and how acquired knowledge could be applied in daily self-management.

## Material and Methods

### Study Design

The study applies a qualitative design with an inductive approach whereby people’s perceptions and experiences are interpreted in the most unconditional way. The study is part of a larger project carried out by our research team, individuals with T1D participated in a three-day diabetes sports camp, including theoretical and practical education (12). Informed consent was collected before the study, which was approved by the regional ethical review board in Uppsala, Sweden (DNR: 2012/159).

### The Context of the Diabetes Sports Camps

In all, eight diabetes sports camps with a total of 105 participants were held. The participants were between 16 and 70 years of age, and all performed PE ≥3 sessions per week. During the camp, all participants received general education and personalized feedback by two physicians and a dietician. The training contained information on insulin adjustments and carbohydrate intake before, during and after PE. The participants practiced carbohydrate counting for all meals. Various types of sports were done one to two times daily. After each training session, the participants received individualized feedback based on downloaded glucose data from real-time Continuous Glucose Monitoring (CGM) devices and insulin doses from Continuous Subcutaneous Insulin Infusion (CSII) pumps or records of doses administered by Multiple Daily Injections (MDI) devices. Assessment of self-perceived knowledge regarding insulin and carbohydrate adjustments related to exercise was performed before and immediately after the camp.

### Sample and Recruitment for Interviews

Interviews were conducted 2–2.5 years after completion of the sports camps. The participants were interviewed about their experiences. A strategic sample procedure was chosen to obtain participants who provided a range of perceptions and experiences. Variation was sought regarding gender, age and sporting ambition (recreational/elite). It was estimated that between 15 and 20 people needed to participate. Of the 19 people who were asked to participate in the interview, 15 agreed to do so. Interviews were conducted by telephone.

### Data Collection and Analysis

The principal interviewer, a nurse with experience of T1D, did not attend any of the diabetes sports camps. Semi-structured telephone interviews were conducted, starting with open-ended questions. A pilot interview was undertaken to test and develop the interview guide, which focused on the participants’ experiences and whether increased knowledge affected their diabetes self-management in everyday life. Some questions concerned the general perception of having T1D adjacent to PE. The interviews were recorded digitally and then transcribed verbatim. According to Graneheim and Lundman, a descriptive qualitative content analysis was used to analyze data (13). The interview text was read repeatedly to get a sense of the overall content. Meaning units were selected based on the study aim and after that, condensed further while keeping the essence intact. The condensed meaning units were sorted into codes that were still very close to data and limited interpretation of content. The next step was to compare codes and cluster them into categories and main categories, based on similar content and meaning. In this phase, a more interpretative process of abstracting data to a higher level was applied, especially in the following step when formulating overall theme (see an example of the analytic process in **Table 1**). Three authors selected meaning units (SM, VB, CSL) and two authors independently coded the data (SM, CSL). Disagreements in coding were resolved through a consensus approach among all authors until joint decisions were reached. Emerging categories and the overall theme were discussed among all the authors for consistency and to secure thrustworthiness.

**Table 1:** Sample illustration of the analytical process.

Meaning unit	Code	Category	Main Category	Theme
The sports camp gave me incredible joy and relief, to meet other people who are in exactly the same situation as me. I am not ashamed that I have diabetes, I have no problem that it beeps (CGM) (p5)	To meet people who are in the same situation	Strengthened by shared experiences	Learning in a motivation-enhancing environment	Empowered by intertwined theory and practice
The camp included education in carbohydrate counting, for me carbohydrate counting works great! I have been asking for a tool for a long time to help me make decisions (insulin dosage), here and now (p14)	Carbohydrate counting is a tool that helps me to decide an appropriate insulin dose for the meal	Carbohydrate counting	Incorporating of new habits	
When you notice that you are good (blood glucose), then you are super … you can do so much more in the workout, so there really is a difference, you dare to give it all (p3)	With good glucose control during exercise you dare to give it your all	Physical performance	Perception of glycemic control and health related outcomes	

## Results

Fifteen individuals were included, of which nine were females and six males. The median age was 42.5 years (range: 28–63). The mean diabetes duration was 22.1 years (range: 0.5–52). The main characteristics of the participants are described in **Table 2**.

**Table 2:** Study sample characteristics.

Number, n	15
Gender (female/male), n	9/6
Age (years), mean ± SD (range)	41.9 ± 9.2 (28–63)
BMI^1^ (kg/m^2^)	24.5 ± 2.1 (20.4–28.3)
Diabetes duration (years), mean ± SD (range)	22.1 ± 14.9 (0.5–52)
Treatment regimen (CSII^2^/MDI^3^), n	10/5
Total daily insulin dose (IU/kg), mean (range)	0.54 ± 0.1 (0.34–0.84)
HbA1c, pre-camp (NGSP^4^, %), mean (range)	7.7 ± 0.9 (6.0–9.3)
HbA1c, pre-camp (IFCC^5^, mmol/mol), mean (range)	60.3 ± 9.5 (42–78)
HbA1c, 12 mo. post-camp (NGSP^4^, %), mean (range)	7.2 ± 0.8 (5.9–8.8)
HbA1c, 12 mo. post-camp (IFCC^5^, mmol/mol), mean (range)	55.6 ± 8.6 (41–73)

BMI, Body Mass Index^1^; CSII, Continuous Subcutaneous Insulin Infusion^2^; MDI, Multiple Daily Injections^3^; NGSP, Glycohemoglobin Standardization Program^4^; IFCC, International Federation of Clinical Chemistry^5^.

**Figure 1:**
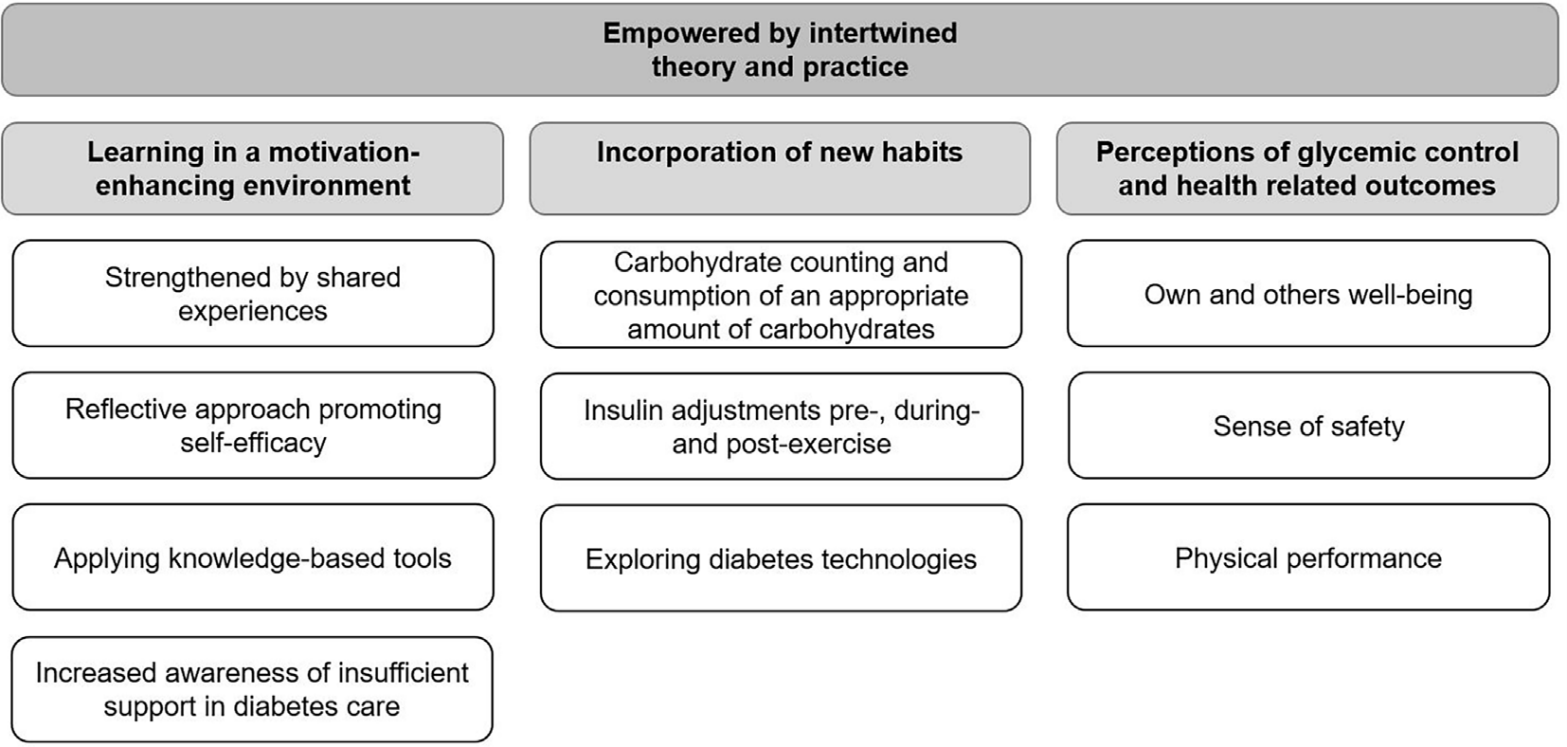
Theme, main categories and categories of the participants’ experiences gained during a sports camp.

The analysis of the interviews resulted in an overarching theme: *Empowered by intertwined theory and practice*. The theme included three main categories: Learning in a motivation-enhancing environment, Incorporation of new habits and Perceptions of glycemic control and health-related outcomes. The three main categories further consisted of a total of ten categories (see **Figure 1**). For those interviewed, a recurring wish was to live a physically active life with good glucose control during exercise, thus enabling them to live a full life together with diabetes. For these participants, mastering their ordinary diabetes self-management in everyday life was not enough. The participants considered the sports camp to be an excellent opportunity to share feelings, ideas, and knowledge and cultivate a sense of belonging with other people with T1D, which was inspiring and motivating. They felt empowered by the camp atmosphere and the supportive environment generated by both participants and experienced HCPs, creating unique conditions to put theory into practice in a safe environment. After the camp, the participants’ general well-being was improved due to incorporating new habits in daily life and physical activity.

Moreover, improvements in glucose control had given them more “energy” during the training sessions and in everyday life. Previously, with the experience of uncontrolled glucose control, they often felt cranky and angry, which also harmed their social life. In the following section, the three main categories are described, followed by exemplifying quotes from participants.

### Learning in a Motivation-Enhancing Environment

#### Strengthened by Shared Experiences

The participants’ shared experience of attending the sports camp gave them a sense of freedom, as they felt there was nothing they had to hide. It did not matter if there was noise from the CSII or CGM-equipment, and it was no big deal if one of the participants reached hypoglycemic glucose levels, as everyone had been through the same thing before, many times. They described pleasure in being able to support others but also in receiving help from other participants experiencing the same problems and issues. They felt a sense of community and that they were like ordinary people and not different or alone. The camp generated the community spirit not just because the participants all had diabetes, but also because they loved PE and different kinds of sports and were eager to accept the challenges of living with T1D.

*The sports camp gave this community, that you have made friends you can contact and ask, how do you solve this, and how do you do this? (p5)*

*One of the absolute best things about the sports camp has been getting a feeling of normality, that you’re not so weird, and that you’re not alone. (p6)*

#### Reflective Approach Promoting Self-Efficacy

Being able to reflect on issues arising when performing physical activities with others in the same situation and accessing knowledgeable HCPs in the area of T1D and PE were described as motivating the participants to adopt a can-do attitude. Theoretical education and assisted analyses of how to make appropriate adjustments of insulin doses and carbohydrate intake before, during and after PE were perceived as new and valuable empowering support. The participants noticed that the methods they learned during the camp worked, which was an essential factor in achieving lasting behavioral change.

*I might have hesitated more, but now I think—no, I actually can!—I will anyway! And I really took home that feeling from the camps. It’s not a question of whether I should do it, it’s just a question of how I should do it. It was a strong feeling that you got! (p6)*

*Eh, and it’s great to get a boost, and you get it at a camp like this, to be reminded—yeah sure! Here’s how to do it … now it’s important to give it your all! (p10)*

#### Applying Knowledge-Based Tools

The participants described receiving a great deal of solid advice during the camp, such as how to adjust insulin doses and how to consume appropriate amounts of carbohydrates in conjunction with PE. They appreciated the constant feedback on how their actions affected glucose regulation during exercise. This feedback was something that the participants found very helpful in fine-tuning their diabetes self-management, both regarding PE and in everyday life after the sports camp.

*To keep this preparatory protocol, and see for a week—what you eat—how much carbohydrate, how much insulin you take and what blood glucose values you have obtained after that (analysis of downloaded glucose data) (p9)*

*Eating the right amount of carbohydrates at the right time, adjusting the insulin doses appropriately, and not like I did before. (p14)*

#### Increased Awareness of Insufficient Support in Diabetes Care

The sports camp increased the participants’ understanding of the strengths and weaknesses of their regular diabetes health care. Most of the participants had experienced that HCPs were not knowledgeable enough in T1D and sport. In general, when asked questions, the caregivers at their diabetes centers had told them to try what suited them best in different situations. HCPs often claimed that, since all individuals are different, it is difficult to give concrete advice. Thus, the participants described often having to learn things through a trial and error strategy. After receiving experiences at the camp, many participants stated that they now expected more of their HCPs.

*There’s a significant lack of knowledge in healthcare—specifically about sports, and I was advised by my doctor, some years ago, to quit sports. So, I haven’t really received any support, either from a diabetes nurse or a doctor. They have no knowledge about sports! I think it’s because they don’t practise sports themselves so much. We haven’t been able to communicate well. (p11)*

### Incorporation of New Habits

#### Carbohydrate Counting and Consumption of an Appropriate Amount of Carbohydrates

Before the sports camp, most participants did not count carbs, but the majority continued with carb counting at home. They said it could be challenging at first, but over time it got easier. Carb counting was considered to make everyday life easier. It helped them choose a proper dose of insulin for the meal and enabled them to compose a meal that provided a sufficient amount of carbohydrates. After starting carb counting, some experienced improved glucose control in everyday life.

*Yes, I count carbs, I didn’t before. So, I learned that at camp, a good way. I weigh pasta and rice after camp. There’s actually a difference. (p9)*

Many participants claimed they did not know enough about the appropriate amount of carbohydrates they needed to consume before, during and after exercise. The participants described how the sports camp had increased their knowledge of how the body works and why a certain amount of carbohydrates may be needed in different situations to maintain stable glucose regulation. The participants also described how they had changed their eating habits in connection with exercise after the camp and how they had started to think more about their diet.

*How to think in connection with exercise was very positive. How to think before, during and after training. I also got to learn about the carbohydrate-mind-set, when you train, to consume carbohydrates, it was a fantastic discovery. (p3)*

*On the days when you exercise, you should eat more, and vice versa. That, along with carbohydrate counting, changed my whole way of managing my diet. I have an incredible improvement in my diabetes control. (p14)*

#### Insulin Adjustments Pre-, During- and Post-Exercise

The participants changed the way they adjusted their insulin doses in connection with exercise and claimed that it had reduced incidences of hypoglycemia after the camp. They reduced their basal insulin before exercise to reduce the risk of hypoglycemia during the workout. Likewise, they understood that they needed less insulin for a given amount of carbohydrates in the meal after exercise due to increased insulin sensitivity.

*We received concrete advice on how we can adjust the insulin dose and to what percentage I have to reduce the insulin dose—and how long before. (p1)*

*But this thing, about being able to reduce the basal insulin and consuming carbohydrates, how I should handle everything and think. Yes, I have learned that! (p12)*

#### Exploring Diabetes Technologies

Consequently, letting all participants use CGM during the sports camp was that most of them became more interested in using technical aids and started to use CGM in everyday life at home. It was not only the opportunity to see their glucose values in real-time that attracted them to using CGM but also that they could download data and subsequently analyze their glucose values. After the camp, some participants started setting aside one day a week to analyze their glucose values for the past week. The evaluation procedure helped them see what had gone well or less well during the previous week, enabling them to fine-tune their insulin dosage and dietary intake. Some participants who used insulin pens during the camp had switched to an insulin pump afterwards, convinced about its benefits by other insulin pump users during the camp.

*The sports camp made me interested in technical aids. I had not received any information about insulin pumps before the camp. I had neither insulin pump nor CGM before the camp. After the camp I’ve been using both an insulin pump and CGM. (p1)*

*CGM has helped me a lot with basal doses. It’s helped me understand what happens during training and after training and so on. (p11)*

### Perceptions of Glycemic Control and Health Related Outcomes

#### Own and Others’ Well-Being

It was stated that knowledge and practice gained at the sports camp also improved diabetes self-management at home after the sports camp. Glucose control had been improved with PE but also in everyday life. Some stated that their quality of life had been affected positively; for example, improved glucose control and general well-being had made them feel more satisfied—something people in their social network had noticed and appreciated.

*I feel that with this knowledge—it’s a different quality of life—absolutely! (p11)*

*It has positively affected them (how improved glucose control affected relatives). I have less mood swings, I am happier and more alert. Again, general health is much better. I’m alert, happy, things are better at work too. I no longer get the dips I had before, with low blood glucose when I’m doing sports. It’s now stable in another way. People in my environment have well received this as a result. My brother also has diabetes, so I gave him the information. He’s also starting to work it out and feel much better with his diabetes. So my parents and siblings and everyone are happy and my partner—they think it’s absolutely brilliant. (p15)*

#### Sense of Safety

The camp leaders’ knowledge and experience in the field of T1D and sports generated trust and made the participants feel safe in the decisions made during camp activities. With knowledgeable HCPs on hand, the participants found carrying out all the different steps themselves a valuable exercise, as it meant they could also perform them at home. Now that they had learned and practiced all the necessary actions while at the camp, their self-confidence and sense of safety/security when performing PE once they got home increased.

*After the camps, I have a completely different sense of security in practicing sports. (p5)*

*My wife probably feels a little safer as well, that I won’t get hypoglycemia and pass out somewhere. Especially if I’m out driving a car. (p10)*

#### Physical Performance

The participants described physical performance during exercise as an important aspect: they wanted to feel good during the workout. They said that before coming to the camp, their bodies did not function as they should during exercise, with a loss of power if blood glucose was too low or high. Glucose excursions meant exercise was not a joyful experience and could negatively affect the rest of their day. After the camp, they said it was easier to achieve more stable glycemic control and that they had more power during their training workouts. The more stable glucose control had a positive effect on their well-being overall.

*If you notice that you are at a good level (blood glucose), then you’re super, you have so much more energy during the workout, you can really give it your all. But as soon as your blood glucose goes down, you can do nothing. Actually it’s also very hard throughout the day then. (p3)*

*It’s more fun to exercise if your blood sugar is good, and that’s that! (p4)*

## Discussion

The current study is the first study to examine experiences from a diabetes sports camp for adults with T1D, where theoretical education was combined with practical exercise and the application of the acquired knowledge in daily self-management. The overall interpretation of the findings is that the intertwined theory and practice during the camps empowered the participants. All of them stated that the main strength was the camp as a whole. Firstly, both the participants and the experienced HCPs had a great interest in practicing different sports. Secondly, the participants and HCPs worked together as a team during the camp with both parties taking part in theoretical education and the practical execution of the exercise.

An important factor in obtaining lasting behavioral changes in an individual’s self-management routine is to do what is to be learned, i.e. hands-on skills training (14). During the sports camp, the participants performed all adjustments with guidance from the HCPs. The participants’ autonomy, self-efficacy and self-management behavior were improved *via* theoretical education and practical execution as a learning principle. It has been shown that patients experience deficiencies due to the gap between theoretical diabetes education and practical implementation of the same (15). The fear of PE-associated hypoglycemias may, for some, mean that they do not even dare to try new methods. The camp provided a safe and secure environment, which was an essential aspect in terms of giving the participants enough confidence to try new methods, such as increasing insulin doses before and during high-intensity anaerobic PE.

The participants said they appreciated the continuous feedback from the physicians, dietitians, diabetes nurses and other camp attendees during and after every workout. Any adjustments to insulin and diet were repeatedly evaluated and readjusted if necessary before the next training session. This continuous evaluation and feedback during and after PE was crucial for learning and would not have been possible during the camps without CGM. Dyck et al. (16) reported that of all the education tools (classroom teaching, real-time CGM, supervised exercise etc.) to learn glucose responses during and after exercise; it was real-time CGM that was considered the most valuable tool for understanding and improved knowledge. Our study confirms this. The participants stated that having CGM improved their knowledge of the importance of adjusting insulin and carbohydrate intake in connection with PE. The overall goal of the sports camps was for every participant to reach glycemic control during exercise. The fact that they were able to achieve stable glucose levels while performing PE was important in increasing self-efficacy and a lasting change in self-management behavior. It has been shown that repeatedly failing to achieve glucose target levels within target ranges is demoralizing and counteracts the participants’ initial feelings of motivation and empowerment (17).

Studies have shown that the participants themselves constitute an important factor for the development autonomy, self-efficacy and self-management in diabetes camps (18–20). The camp members can become advisors and role models, which in turn facilitates the individual’s acceptance of their condition and leads to improved engagement in self-management attitudes (19). This is in line with the findings in our study, where all participants experienced that meeting other individuals with T1D was inspiring, motivating and rewarding. The participants stated that they were affected by the other participants’ attitudes, thoughts, values and reactions. They adopted more of a can-do-attitude, the question being not whether or not they should do something, but simply how they should do it.

In our study, all camp attendees participated in lectures and discussions, where the aim was to understand the physiology during and after exercise. They were also presented with a rationale for why insulin and/or carbohydrate intake should be adjusted in different situations to achieve stable glucose regulation. The goal was to enable them to make appropriate choices about insulin and carbohydrates in connection with different kinds of PE and thus help them achieve the desired result of proactive self-management at home, after the camp. In the interviews, the participants said they had incorporated many of these new habits in daily life, and the main reason they were maintaining their new diabetes self-management behavior was that they noticed it was working. It is known that for new self-management behaviors to be permanent, it is crucial for individuals that the result is in line with the intended outcome (17). The participants reported that their glucose control had improved after the camp and that the incidence of hypoglycemia decreased in connection with both exercise and everyday life. They also said that glycemic control during their training sessions was often reflected in their mood even afterwards. If glucose control was poor during exercise, the rest of the day could feel worse as a result. Additionally, overall improved glucose control made them happier and more satisfied with life in general, which relatives and friends also confirmed.

In the current study, all HCPs had extensive experience in the field of T1D and sports. Previous studies have shown that, during a diabetes camp, a well-trained staff of HCPs is absolutely crucial to achieving an autonomy-supportive environment (18, 19). Our results showed that the majority of the participants’ felt that their regular HCPs did not have sufficient knowledge within the field of T1D and sports to be able to provide them with guidance on appropriate insulin adjustments and carbohydrate intake associated with PE. These findings are in line with other studies that have examined the level of knowledge of health professionals in the field of exercise (10, 21, 22). This is a major problem in regular diabetes care. In order for clinicians to give advice in the area of T1D and PE, they must have a certain understanding of the metabolic response that occurs during different types of PE and what requirements this places on both insulin adjustments and carbohydrate requirements. To arrange sports camps similar to the one in this study requires large resources, both in working hours and financially. However, the most important factor for the success in these sports camps was probably that all HCPs were well educated within the field of T1D and PE and the participants were eager to learn. This constellation of people can also be obtained in web-based education programs where experiences also can be exchanged between patients. Web-based education has been shown to improve knowledge, self-efficacy and self-management behaviors in health care (23–25). The most effective way to reach higher levels of knowledge about PE and T1D in both patients and HCPs may be to use web-based solutions in regular diabetes care (24, 26, 27).

Our results show that the sports camps are educational and rewarding for the patients, but could be considered costly. Another feasible solution, however, is to organize national diabetes sports camps annually where patients and HCPs from diabetes teams are invited. This approach would gradually increase the level of competence of both patients and HCPs.

### Limitations

A limitation of this study is that selection bias could exist because the study participants were not randomly selected from the TID population. Moreover, the participants themselves expressed their interest in participating in the camps. The inclusion criterion for participating at the camps was that the participants would exercise ≥3 workout sessions/week, which probably yielded a relatively homogeneous group of participants who were determined to improve glycemic control. Thus, the participants may not be a representative sample of adult subjects with T1D who regularly practice physical activity in general.

## Conclusion

The experiences from a dedicated diabetes sports camp focusing on achieving good glucose control during daily training sessions indicate that this kind of complimentary support in diabetes care can empower individuals to become more independent and autonomous and thus able to live a full life with diabetes and physical activity. The positive atmosphere that arose during the sports camps was primarily due to the fact that both HCPs and the patients had common interests—T1D and PE. These people could also meet and exchange experiences in other contexts. The post-pandemic digital development has provided new opportunities for web-based solutions. Thus, web-based education packages in the area of T1D and PE could be used to increase the level of knowledge for both patients and healthcare professional—physicians, dietitians and nurses in diabetes care.

## Data Availability Statement

The raw data supporting the conclusions of this article will be made available by the authors, without undue reservation.

## Ethics Statement

The studies involving human participants were reviewed and approved by Regional ethical review board in Uppsala, Sweden (DNR: 2012/159). The patients/participants provided their written informed consent to participate in this study.

## Author Contributions

SM and CS-L conceived and designed the research. PA and JJ participated in the planning of the study. VB conducted all interviews. SM, CS-L, PA, and JJ conducted a descriptive qualitative content analysis of collected data. SM wrote the manuscript. CS-L, PA, JJ, and VB reviewed the manuscript. All authors contributed to the article and approved the submitted version.

## Funding

The study was funded by an unrestricted grant from Novo Nordisk AS, Bagsværd, Denmark. The funder was not involved in the study design, collection, analysis, interpretation of data, the writing of this article or the decision to submit it for publication.

## Conflict of Interest

The authors declare that the research was conducted in the absence of any commercial or financial relationships that could be construed as a potential conflict of interest.

## Abbreviations

A1c, Glycated Hemoglobin; BMI, Body Mass Index; CGM, Continuous Glucose Monitoring; CSII, Continuous Subcutaneous Insulin Infusion; IFCC, International Federation of Clinical Chemistry; MDI, Multiple Daily Injections; NGSP, National Glycohemoglobin Standardization Program; PE, Physical Exercise; T1D, Type 1 Diabetes.
